# Spatiotemporal Modeling and Intelligent Recognition of Sow Estrus Behavior for Precision Livestock Farming

**DOI:** 10.3390/ani15192868

**Published:** 2025-09-30

**Authors:** Kaidong Lei, Bugao Li, Hua Yang, Hao Wang, Di Wang, Benhai Xiong

**Affiliations:** 1 College of Information Science and Engineering, Shanxi Agricultural University, Jinzhong 030801, China; leikaidong@caas.cn (K.L.); bugaoli1@sxau.cn (B.L.); wangdi@sxau.cn (D.W.); 2 College of Engineering, China Agricultural University, Beijing 100083, China; wanghao@cau.cn; 3 State Key Laboratory of Animal Nutrition, Institute of Animal Sciences, Chinese Academy of Agricultural Sciences, Beijing 100193, China; xiongbenhaii@caas.cn

**Keywords:** sow estrus, video analysis, CNN + LSTM, 3D-CNN, CNN + TCN, deep learning, precision livestock farming

## Abstract

Accurate recognition of estrus behavior in sows is of great importance for achieving scientific breeding management, improving reproductive efficiency, and reducing labor costs in modern pig farms. However, due to the evident spatiotemporal continuity, stage-specific changes, and ambiguous category boundaries of estrus behaviors, traditional methods based on static images or manual observation suffer from low efficiency and high misjudgment rates in practical applications. To address these issues, this study follows a video-based behavior recognition approach and designs three deep learning model structures: (Convolutional Neural Network combined with Long Short-Term Memory) CNN + LSTM, (Three-Dimensional Convolutional Neural Network) 3D-CNN, and (Convolutional Neural Network combined with Temporal Convolutional Network) CNN + TCN, aiming to achieve high-precision recognition and classification of four key behaviors (SOB, SOC, SOS, SOW) during the estrus process in sows. In terms of data processing, a sliding window strategy was adopted to slice the annotated video sequences, constructing image sequence samples with uniform length. The training, validation, and test sets were divided in a 6:2:2 ratio, ensuring balanced distribution of behavior categories. During model training and evaluation, a systematic comparative analysis was conducted from multiple aspects, including loss function variation (Loss), accuracy, precision, recall, F1-score, confusion matrix, and ROC-AUC curves. Experimental results show that the CNN + TCN model performed best overall, with validation accuracy exceeding 0.98, F1-score approaching 1.0, and an average AUC value of 0.9988, demonstrating excellent recognition accuracy and generalization ability. The 3D-CNN model performed well in recognizing short-term dynamic behaviors (such as SOC), achieving a validation F1-score of 0.91 and an AUC of 0.770, making it suitable for high-frequency, short-duration behavior recognition. The CNN + LSTM model exhibited good robustness in handling long-duration static behaviors (such as SOB and SOS), with a validation accuracy of 0.99 and an AUC of 0.9965. In addition, this study further developed an intelligent recognition system with front-end visualization, result feedback, and user interaction functions, enabling local deployment and real-time application of the model in farming environments, thus providing practical technical support for the digitalization and intelligentization of reproductive management in large-scale pig farms.

## Simple Summary

Estrus detection in sows is a key challenge in precision livestock farming, as traditional methods are labor-intensive, subjective, and prone to errors. To address this challenge, this study developed and compared three deep learning models (Convolutional Neural Network combined with Long Short-Term Memory, CNN + LSTM), (Three-Dimensional Convolutional Neural Network, 3D-CNN), and (Convolutional Neural Network combined with Temporal Convolu-tional Network, CNN + TCN) based on behavioral video sequences, systematically evaluating their classification performance across multiple estrus-related behaviors. The results show that the CNN + TCN model performs best in terms of recognition accuracy and stability, particularly suitable for behavior recognition tasks with multi-stage and strong temporal characteristics. Based on this, an intelligent recognition system with front-end display and interactive functions was further developed, enabling real-time monitoring and assisted decision-making of sow estrus behaviors, thereby providing a feasible path for the construction of smart pig farms.

## 1. Introduction

In China, Precision Livestock Farming has been developing rapidly [1,2,3,4,5]. In large-scale pig production systems, the reproductive efficiency of sows is not only related to the selection and breeding of pigs, but also directly affects the economic returns of the entire breeding chain of breeding pigs [6]. As a core component of reproductive management, the accurate identification of sow estrus behavior is essential for ensuring optimal mating timing and improving conception rates [7,8,9]. However, most farms still rely on traditional methods such as manual observation or the standing-back pressure test to determine estrus status. These conventional approaches are experience-dependent, highly subjective, labor-intensive, and inefficient, making them inadequate to meet the modern pig farm’s demand for efficient and precise management. Therefore, developing a high-performance, low-cost, and deployable automatic sow estrus behavior recognition system has become both a critical technical bottleneck and an urgent need in the construction of intelligent livestock farming systems.

The estrus behavior of sows exhibits typical multi-stage dynamic characteristics, generally including key manifestations such as standing still, ears held erect, accepting mounting by boars, and emitting low grunting sounds [10]. The temporal transitions between different behaviors are not clearly defined, their durations vary significantly, and they exhibit high heterogeneity and uncertainty across individuals. This complex temporal evolution makes it difficult for traditional methods—relying on single-frame images or static feature extraction—to accurately capture behavior trajectories and estrus progression trends. More notably, most pig farms are still not equipped with intelligent recognition systems that offer interactivity, practicality, and adaptability for deployment. As a result, farm staff often lack efficient, objective, and continuous behavioral assessment support in actual management, which limits further advancement in the intelligent monitoring of estrus.

In recent years, video-based behavior recognition technology has been gradually applied to the intelligent monitoring of livestock and poultry, achieving certain progress in the recognition of locomotion, feeding, and aggressive behaviors in animals such as cattle, pigs, and chickens [11,12,13,14,15,16]. With the rapid development of artificial intelligence technology, deep learning models such as two-dimensional convolutional neural networks (2D-CNN), three-dimensional convolutional networks (3D-CNN), long short-term memory networks (LSTM), and temporal convolutional networks (TCN) have demonstrated good performance in human behavior recognition [17,18], These related methods have also gradually been introduced into animal behavior modeling research, improving recognition accuracy and model generalization ability. However, most current studies still focus primarily on spatial feature extraction and classification from static images or single-frame videos, with insufficient modeling of behavioral evolution in the temporal dimension and a lack of in-depth analysis of complex spatiotemporal dynamics. In addition, existing research mostly remains at the algorithmic model level and has yet to form systematic solutions integrated with actual farming practices, especially lacking human–machine interaction interfaces that collaborate with recognition models, which restricts the practical application of related achievements in real production environments. Considering the limitations of on-site farming equipment and operational demands, recognition algorithms still require further optimization in terms of lightweight design and real-time responsiveness to meet the comprehensive requirements of smart pig farms for efficiency and deployability.

Although extensive research has been conducted on sow estrus behavior recognition [9,19,20,21,22], there remain technical shortcomings in spatiotemporal modeling accuracy, system integration capability, and practical deployment adaptability. To address these issues, this study proposes an intelligent recognition and interaction system integration method for sow estrus behavior based on video analysis, oriented toward precision livestock farming. The overall design balances the recognition accuracy of algorithmic models with the practicality of system platforms, aiming to realize a closed-loop human–machine collaboration from behavior recognition to decision support. The specific innovations are as follows:

(1) Multi-model construction and system evaluation: Three typical spatiotemporal modeling methods (CNN + LSTM, 3D-CNN, CNN + TCN) are systematically constructed and compared for the first time, with evaluations conducted from the perspectives of temporal sequence modeling capability, behavioral dynamic feature perception, and computational efficiency, providing methodological references for model selection and application deployment in sow estrus behavior recognition tasks.

(2) Multi-category estrus key behavior modeling framework design: Aimed at the behavioral evolution characteristics throughout the estrus process, the framework integrates spatial features and temporal patterns, and extracts the dynamic evolution structure among behaviors through spatiotemporal sequence modeling, enhancing the recognition system’s expressive capacity for complex behavior patterns.

(3) Real-world scenario experiment validation and multi-metric evaluation: Experiments are conducted in representative sow farming scenarios. Using mainstream evaluation metrics such as confusion matrix, F1-score, ROC curves, and AUC values, the models are comprehensively assessed in terms of accuracy, recognition stability, and practical usability, thereby validating the practical value and application potential of the proposed method.

In summary, this study not only conducted an in-depth exploration of spatiotemporal modeling for sow estrus behavior recognition at the methodological level, but also developed a deployable and scalable intelligent platform in terms of system integration and practical application, promoting the in-depth application of precision farming technologies in estrus management scenarios on pig farms.

## 2. Materials and Methods

### 2.1. Experimental Site and Data Acquisition

To achieve sow estrus detection based on machine vision, this study designed and implemented a multimodal behavior detection system integrating a bionic boar module, an intelligent mobile chassis, bionic odor and sound simulation devices, and a PC-based recognition terminal. The system software environment is based on Python version 3.7. The system aims to induce natural estrus responses in sows by simulating boar appearance, behavior, and olfactory stimuli, and to realize high-precision behavior modeling and estrus determination through video imagery and behavioral response features.

**Figure 1:**
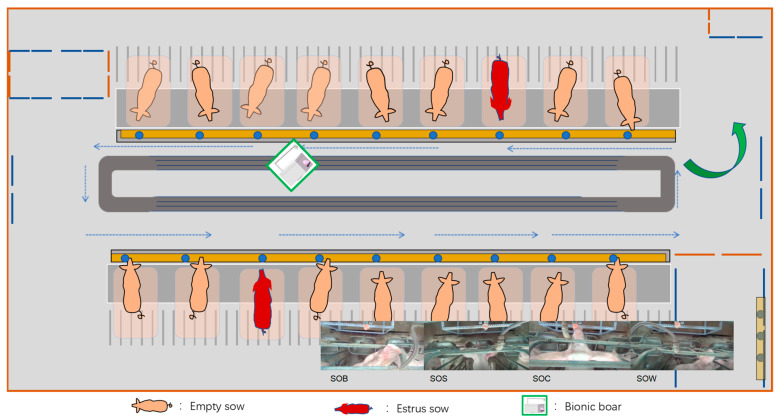
Experimental design and overall overview diagram as well as behavioral overview.

This study conducted behavior acquisition and robotic application experiments at three large-scale sow breeding farms in Shandong, Chongqing, and Shanxi from July 2019 to December 2020 and from December 2024 to August 2025. The experimental subjects were post-weaning sows with 2 to 3 parities. In total, 105 groups of sow behavior data were collected. Based on the ethological characteristics of estrus behavior, a bionic boar interaction device was designed and developed to intelligently detect the estrus state of sows. The device features a 1:1 scale 3D-printed boar head as its core structure, simulating key behaviors such as sniffing and physical contact commonly exhibited by boars during estrus detection. An ultrasonic module capable of controlled release of androstenone was integrated to enhance the sow’s neuro-endocrine response, thereby improving the stimulation effect and behavior response rate. The bionic boar device incorporates three simulation functions—sound, odor, and tactile feedback—and introduces a “contact window” mechanism to strengthen physical interaction, thereby promoting the natural expression of estrus behavior. It can actively simulate low-frequency vocalizations and olfactory cues of boars, and directionally release sex pheromones such as androstenone to induce typical behavioral responses in sows, including standing immobile, approaching, and exploratory actions. Based on these responses, combined with machine vision-based behavior recognition techniques, real-time perception and automatic identification of estrus behaviors in non-pregnant sows were achieved. Furthermore, a dynamic perception model of estrus state was constructed by analyzing behavioral responses under hormonal stimulation from the bionic boar. Interaction patterns between the sow and the device were also modeled and intelligently identified. Figure 1 provides an overview of the entire framework of this study. This approach enables precise determination of the estrus phase and assists in identifying the optimal timing for mating, thereby providing crucial support for the construction of an intelligent sow reproductive management system and significantly improving reproductive efficiency and the level of intelligent management.

### 2.2. Behavior Classification Scheme and Annotation Protocol

To systematically model the behavioral evolution of sows during the estrus cycle, this study established a four-category classification system for key estrus-related behaviors, grounded in biological evidence and feasible for behavior recognition. This system encompasses representative behavioral characteristics observed during the estrus phase and is defined as follows: S-O-B: Exploratory behaviors such as bar-biting exhibited by sows during the estrus period. S-O-S: A clear standing reflex, typically regarded as a crucial indicator of mating receptivity. S-O-C: Active contact by the sow with the bionic boar’s nose, indicating approach and acceptance behavior. S-O-W: Noticeable and large-amplitude head-swinging movements, A reaction representing restlessness. This classification system was developed based on ethological principles and practical experience in farm environments. It effectively captures the main behavioral transition patterns of sows during estrus and exhibits strong observability, temporal consistency, and inter-class separability.

To ensure accurate and temporally aligned labeling of training data, a frame-by-frame annotation strategy was adopted for the raw video footage, enabling precise localization and consistent coding of each behavior along the time axis. The total number of frames for each behavior category is as follows: 31,835 for SOB, 15,599 for SOC, 33,513 for SOS, and 38,963 for SOW. Due to a certain degree of class imbalance in the final constructed dataset, data augmentation strategies—including temporal cropping, mirroring, and random perturbation—were employed during the model training phase to improve sample distribution balance and enhance the model’s generalization capability.

### 2.3. Data Preprocessing and Dataset Construction

After completing the behavior annotation, systematic data preprocessing and dataset construction were performed to meet the temporal-structured input requirements of deep learning models.

First, considering the strong dependence of estrus behavior recognition on temporal continuity and contextual information, this study adopted a fixed-length slicing combined with a sliding window strategy to segment all annotated behavior clips. During the dataset construction phase, to enhance the model’s generalization ability and ensure scientific evaluation, a stratified random partitioning method was employed to divide all samples into training (60%), validation (20%), and test (20%) subsets. Throughout the partitioning process, the proportion of each behavior category was strictly maintained across the three subsets to avoid model bias caused by class imbalance, thereby ensuring the stability of model training and the reliability of evaluation metrics.

As a result, the dataset constructed through the above preprocessing pipeline exhibits strong temporal integrity, structural consistency, and category balance, providing a solid data foundation for efficient training and stable inference of the subsequent deep behavior recognition models.

### 2.4. Spatiotemporal Modeling Architectures

#### 2.4.1. CNN + LSTM Model

Among the three behavior recognition models constructed in this study, the CNN + LSTM model adopts a hierarchical modeling architecture that decouples spatial feature extraction from temporal sequence modeling, aiming to recognize key behavioral sequences during the sow estrus cycle. Specifically, the model first employs a lightweight two-dimensional convolutional neural network (CNN) to extract spatial information from individual frames, and then feeds the frame-level features into a multi-layer bidirectional LSTM for temporal modeling and classification. In the spatial feature extraction stage, the model uses torchvision.models.resnet18 as the backbone network. The final classification layer is removed, retaining only up to the Global Average Pooling (GAP) layer, resulting in an output feature dimension of 512. Input videos are uniformly segmented into continuous sequences of 32 frames, and each frame is passed through the ResNet backbone for forward inference to extract semantic feature vectors. The final output is a feature sequence of shape [B, T, 512], where B denotes the batch size and T = 32 represents the number of time steps.

Subsequently, the feature sequence is fed into a stacked two-layer bidirectional LSTM (BiLSTM) network for temporal modeling, with each hidden layer consisting of 128 units. The output sequence from the LSTM is aggregated along the temporal dimension using mean pooling to generate a fixed-dimensional global behavior representation. This representation is then passed through a fully connected layer with Dropout (set to 0.5) and LayerNorm to perform fore-class behavior classification and output the corresponding behavior probability distribution. The classification layer employs a Softmax activation function (based on Python version 3.7), producing a four-dimensional vector that represents the confidence scores for the fore behavior categories. During training, the model uses the cross-entropy loss function (nn.CrossEntropyLoss) and is optimized with the AdamW optimizer (learning rate set to 1 × 10^−4^, with weight decay). A learning rate scheduler (ReduceLROnPlateau) is applied to dynamically reduce the learning rate when the validation performance plateaus. To improve training stability and computational efficiency, automatic mixed precision (AMP) via torch.cuda.amp and GradScaler is enabled, which helps reduce GPU memory usage and accelerates model convergence.

**Figure 2:**
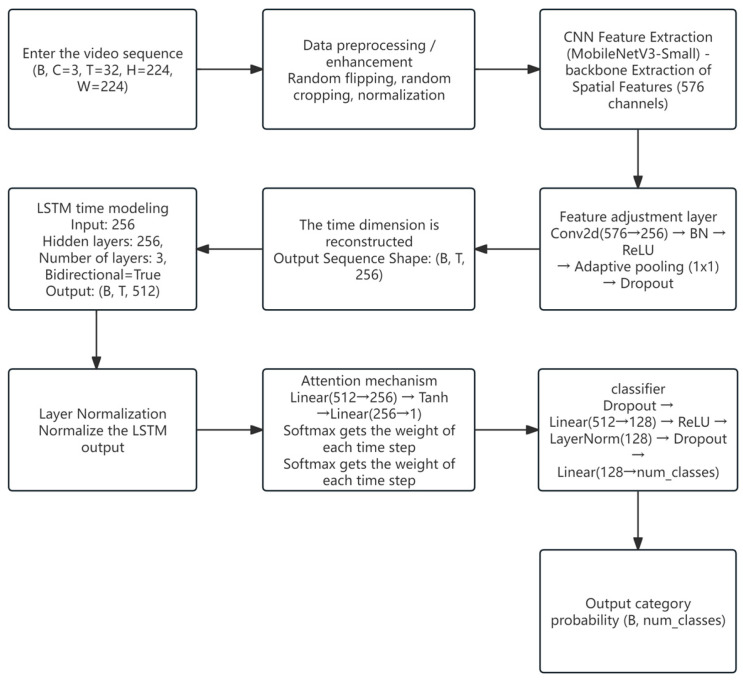
Overview diagram of the LSTM model structure.

In addition, the data augmentation phase incorporates random flipping and random cropping strategies, effectively enhancing the model’s robustness to variations in postures and background interference across different scenarios. The overall architecture is designed with a balanced consideration of training efficiency and temporal behavior perception, making it well-suited for the recognition of estrus behaviors in sows characterized by stage-specific and continuous patterns [23]. The structure of the LSTM model is shown in Figure 2.

#### 2.4.2. 3D-CNN Model

The 3D-CNN model (C3DModel) adopts an end-to-end three-dimensional convolutional architecture to simultaneously extract spatial and temporal dynamic features from sow behavior video segments. The model architecture is based on the classical C3D structure and is modified through a series of structural prunings and optimizations to balance behavior representation and computational efficiency under livestock farming scenarios. At the input stage, each video clip is uniformly processed into 32 consecutive frames (size 224 × 224, RGB three channels), and reshaped into a 5D tensor of shape [B, C = 3, T = 32, H = 224, W = 224] as the input to the model. For feature extraction, the model is constructed with four convolutional blocks: the first layer uses Conv3D (3 → 128), followed by Conv3D (128 → 256), Conv3D (256 → 512), and Conv3D (512 → 512). Each convolutional layer is followed by BatchNorm, a ReLU activation function, and a spatial or spatiotemporal MaxPooling operation. Finally, AdaptiveAvgPool3d ((1, 1, 1)) is applied to obtain a compact feature representation of the video block.

The feature vector is flattened and passed through a three-layer fully connected network: Linear (512 → 1024), Linear (1024 → 512), and Linear (512 → 5), with Dropout (0.5/0.3) and ReLU activation functions inserted between layers to prevent overfitting. The final output is a 5-dimensional softmax probability vector used to predict which class of estrus-related key behavior the video clip belongs to. During training, the loss function is set to CrossEntropyLoss, and the optimizer is AdamW with an initial learning rate of 1 × 10^−4^. The learning rate is scheduled using ReduceLROnPlateau (factor = 0.5, patience = 10), and early stopping is applied (patience = 30, min_delta = 0.005) to avoid overfitting. To improve training efficiency and stability, PyTorch’s GradScaler (based on Python version 3.7) is introduced for mixed precision training (AMP), and gradient accumulation and gradient clipping (clip_grad_norm_) are used to handle the memory pressure caused by multi-frame high-dimensional inputs.

**Figure 3:**
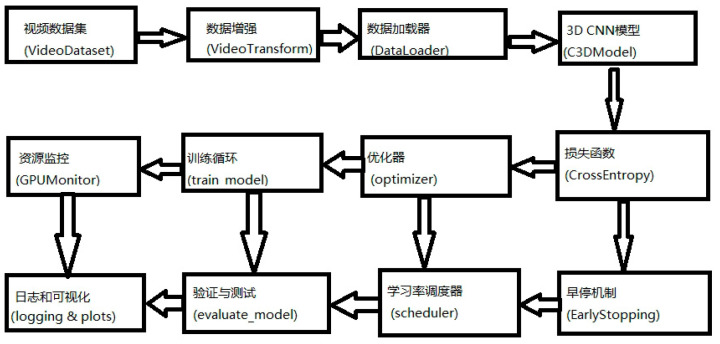
Model framework diagram for video data processing.

In addition, multiple data augmentation strategies are applied at the model input stage, including sliding window random clipping, temporal reversal, spatial flipping, and random cropping. During data loading, frame number completion and channel rearrangement are also implemented to ensure the generalization ability of video clips under varying scales, postures, and backgrounds. Figure 3 presents the framework of the 3D-CNN model for video data processing.

This 3D-CNN model possesses a complete spatiotemporal joint modeling capability, performs stably in behavior recognition tasks with strong continuity and pronounced temporal dependency, and is suitable for clip-level recognition of estrus behaviors such as sow standing still, walking, and mounting. It also provides essential high-dimensional spatiotemporal feature extraction capability for the overall model system.

#### 2.4.3. CNN + TCN Model

The CNN + TCN model (Temporal Convolutional Network) integrates the spatial feature extraction capability of 2D convolutional neural networks with the sequence modeling capacity of dilated convolutions, enabling effective recognition of multi-stage estrus behaviors in videos. By decoupling the modeling of spatial and temporal information, this model enhances sensitivity to behavioral evolution and is suitable for identifying behavior sequences with stage continuity and temporally ambiguous boundaries [24].

**Figure 4:**
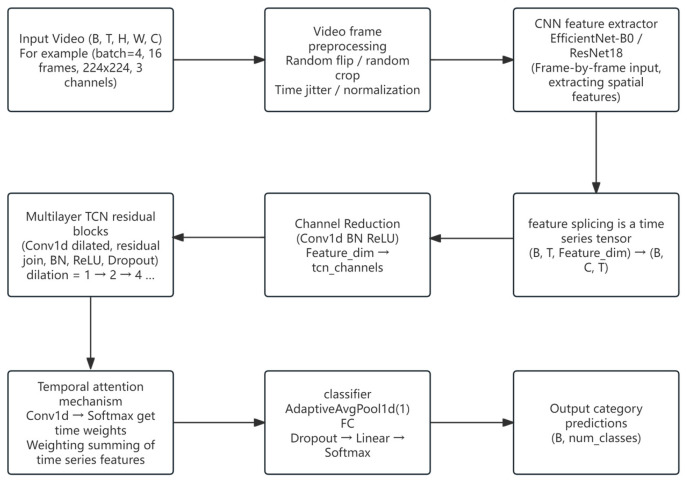
Overview diagram of the TCN model structure.

In the spatial feature extraction stage, the backbone network was configured as EfficientNet-B0 by default, while ResNet18 was additionally tested for comparison. The two networks output 1280- and 512-dimensional features, respectively, which were then compressed to 64 dimensions using a 1 × 1 convolution. Following the backbone CNN, a 1 × 1 convolutional channel compression layer is applied to reduce the frame-level feature dimension to 64, followed by BatchNorm and ReLU activation for spatial encoding. The temporal modeling component consists of multiple stacked TCN residual blocks. Each TCN residual block contains two dilated convolutions (with dilation rates of 1, 2, and 4), employs ReLU activation, and incorporates Dropout (0.2) along with residual connections to ensure effective information flow through deep layers. This structure enables the modeling of both short-term and long-term dependencies, making it suitable for multi-stage behavior modeling scenarios. After the TCN output, a lightweight temporal attention module is added, which utilizes 1D convolution and Softmax to learn the weight distribution over time steps, thereby achieving dynamic weighting of key behavior moments. For classification, the model applies adaptive average pooling to extract a global representation of the sequence, followed by Dropout (0.2), and a fully connected layer outputs the multi-class behavior prediction probabilities. The training process uses CrossEntropyLoss as the loss function and AdamW as the optimizer, with an initial learning rate of 1 × 10^−4^. The learning rate is dynamically adjusted using the ReduceLROnPlateau strategy, and early stopping is supported to prevent overfitting. In addition, the data augmentation pipeline integrates spatial random cropping, horizontal flipping, and temporal jittering to enhance the model’s adaptability to different sow postures and background environments. In practical testing, this model demonstrated good performance in recognizing standing and head-shaking behaviors, and its lightweight design and high deployability make it suitable for deployment on edge devices or embedded systems. Figure 4 presents the Overview diagram of the TCN model structure.

### 2.5. Model Training and Evaluation Strategy

To ensure the comparability, fairness, and scientific validity of performance comparisons among the three sow estrus behavior recognition models (CNN + LSTM, CNN + TCN, and 3D-CNN), this study designed a unified training and evaluation strategy. All experiments were conducted on a workstation equipped with an NVIDIA GeForce RTX 4080 Super GPU, 128 GB RAM, and an Intel CPU running Windows 10. The software environment was based on Python 3.8 with deep learning frameworks installed accordingly. The configurations were standardized across loss functions, optimizers, learning rate scheduling, and evaluation metrics, aiming to comprehensively assess the models’ classification accuracy, robustness, and deployment efficiency.

During the training phase, the cross-entropy loss function (CrossEntropyLoss) was uniformly applied to measure the discrepancy between the model’s predicted distribution and the ground truth labels in multi-class classification tasks. The optimizer was set to AdamW, which incorporates weight decay to effectively improve model generalization. Additionally, a model checkpointing mechanism was enabled to automatically save the model weights corresponding to the best validation performance, ensuring that the optimal model version is used in the final evaluation stage. To improve training efficiency, all models employed automatic mixed precision training (AMP) and gradient scaling (GradScaler), effectively reducing GPU memory consumption while maintaining numerical accuracy, thereby accommodating the processing needs of high-dimensional video input data.

In the evaluation phase, model performance was comprehensively quantified across multiple dimensions. Accuracy was used to assess the overall correctness of classification. Precision, recall, and F1-score were employed to characterize the recognition capability of the model for each class, particularly suitable for scenarios with class imbalance. The confusion matrix provided an intuitive display of misclassification between classes and blind spots in recognition, offering a basis for error analysis. Under the above configuration, due to the large number of parameters, the 3D-CNN model required approximately 5 min per training epoch, while the CNN + LSTM and CNN + TCN models took approximately 2.5 to 3 min per epoch. All models were trained and evaluated using the same training/validation/test dataset split and evaluation criteria, ensuring the scientific rigor of the experimental conclusions and the validity of horizontal comparisons.

In summary, the unified training and evaluation strategy balances model accuracy, training stability, and deployment friendliness, providing a reliable experimental basis and evaluation framework for model performance comparison, system integration, and subsequent deployment.

### 2.6. Interactive System Development and Integration

To achieve intelligent estrus detection operations, the above modules are integrated. The specific functional modules include: a video upload and preprocessing module, where users can upload designated video segments via the system interface, and the system performs preprocessing operations such as frame splitting and size normalization; a real-time recognition module, where front-end video streams or uploaded samples can be fed into the deployed model in real time for behavior prediction and probability output; a behavior visualization and annotation display module, where recognition results are presented on a timeline, marking each behavior segment and its corresponding confidence score, while also supporting start-end time positioning and highlight display; and a user interaction feedback module, where farm personnel can revise, label, or comment on recognition results to assist in constructing a closed-loop feedback system for behavioral data. The platform maintains an overall response time within 1 s, with model inference time per video segment approximately 100–180 ms, achieving near real-time behavior recognition and interactive feedback. The integration and modular design of the system not only meet the practical application needs of estrus behavior detection tasks, but also provide a sound extension interface for future model upgrades and functional expansions (such as linked alerts and breeding plan formulation), enabling further evolution towards an “intelligent reproduction management platform” [25,26].

## 3. Results

### 3.1. CNN + LSTM Model Performance

To further verify the effectiveness of fusion modeling based on two-dimensional images and time series, the CNN + LSTM model was selected for behavior classification experiments, with a comprehensive assessment of its learning trends during training and final recognition performance.

#### 3.1.1. Training and Validation Process 

**Figure 5:**
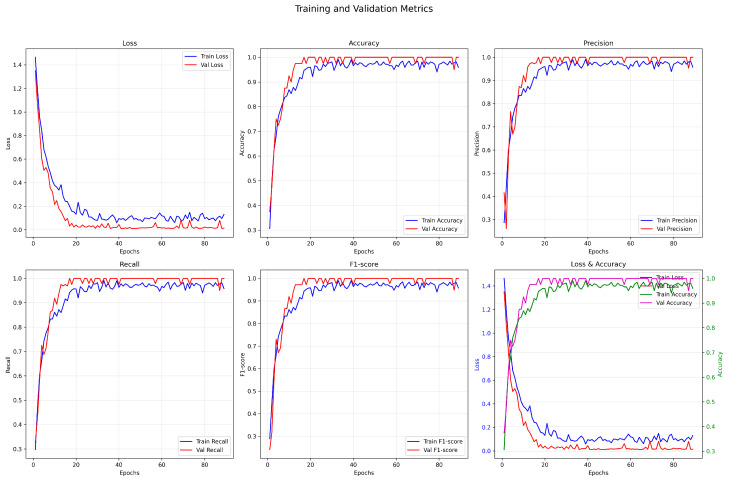
Training and validation index change curve.

As shown in the training and validation metric curves (Figure 5), the model converged rapidly in the early training stage, with the training loss dropping significantly to below 0.2 after approximately 15 epochs, and continuing to decrease gradually to around 0.1. The validation loss exhibited minor fluctuations, with an overall trend consistent with the training loss, indicating no significant overfitting. Regarding key metrics such as accuracy, precision, recall, and F1-score, the CNN + LSTM model showed a substantial improvement after 20 epochs and remained stable in subsequent training. Specifically, the training accuracy eventually stabilized around 0.97, while the validation accuracy reached 0.99; the validation F1-score remained above 0.98, demonstrating the model’s stable and excellent classification capability across different behaviors. Moreover, the combined “Loss & Accuracy” curve indicates a stable training process, with small discrepancies among metrics, reflecting good generalization performance.

#### 3.1.2. Confusion Matrix Analysis

**Figure 6:**
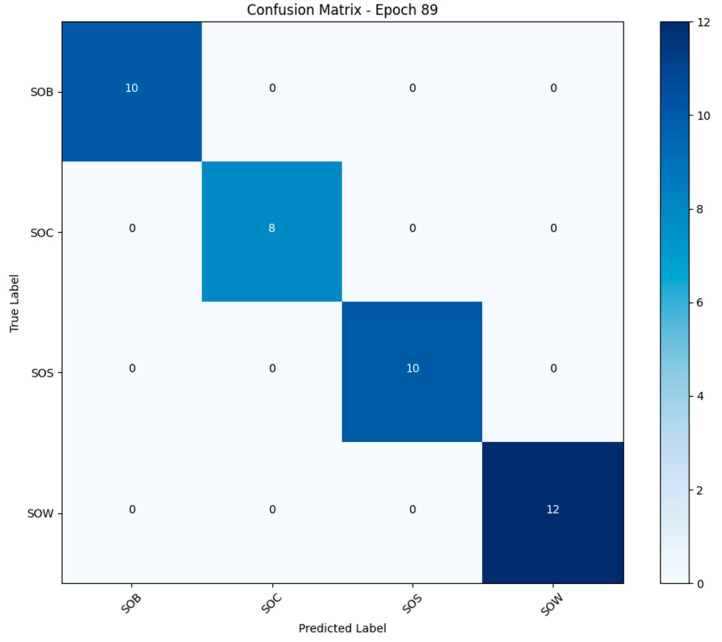
Confusion matrix analysis diagram.

At the 89th epoch of training, the model’s classification results on the test set are shown in Figure 6. According to the confusion matrix, the model achieved 100% recognition accuracy for all four behavior categories (SOB, SOC, SOS, SOW), with no misclassified samples. Specifically, all 10 samples of the SOB class were correctly classified; all 8 samples of the SOC class were correctly classified; all 10 samples of the SOS class were correctly classified; and all 12 samples of the SOW class were correctly classified.

The above results indicate that the CNN + LSTM model possesses strong discriminative capability for different categories of behavioral features and can effectively capture the coupling relationship between temporal dynamics and spatial representation features.

#### 3.1.3. ROC Curve and AUC Evaluation

**Figure 7:**
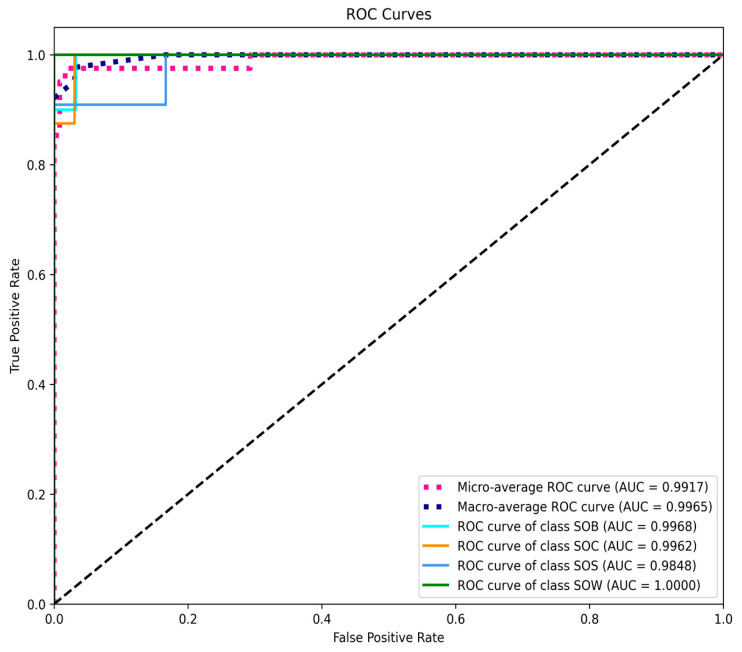
ROC Curve and AUC evaluation.

To further evaluate the model’s classification capability, ROC curves were plotted, and AUC values were calculated for the four behavior categories (Figure 7). Specifically, the micro-averaged ROC curve yielded an AUC of 0.9917, and the macro-averaged ROC curve achieved an AUC of 0.9965. All individual categories attained AUC values exceeding 0.98, with the SOW category reaching 1.0000, demonstrating outstanding performance. As shown in this figure, the ROC curves for all categories closely approach the top-left corner, indicating excellent discrimination between positive and negative samples and reflecting high classifier reliability. Notably, the perfect AUC score of the SOW category suggests that its behavioral features were thoroughly learned by the network.

### 3.2. 3D-CNN Model Performance

To evaluate the classification performance of the constructed model in the task of sow estrus behavior recognition, this study trained and tested a deep 3D-CNN model, with key performance metrics systematically recorded throughout the training and validation processes. The detailed results for each metric are presented below:

#### 3.2.1. Training and Validation Process

(1) Accuracy and Loss Trends

**Figure 8:**
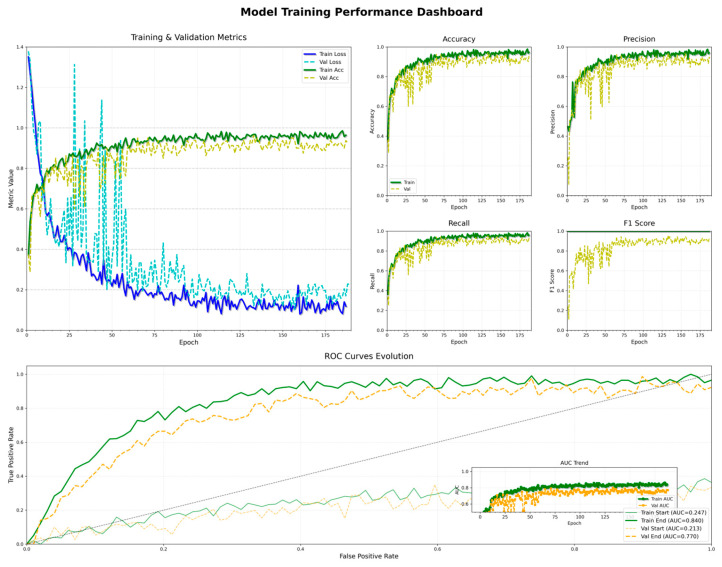
D-CNN Model training diagram.

The loss and accuracy curves during the training and validation of the model are shown in Figure 8. The training loss dropped rapidly in the early stages—from approximately 1.3 to around 0.1—indicating fast convergence and strong learning capability. The validation loss eventually stabilized at around 0.2 with minimal fluctuation, suggesting good generalization performance on unseen data. In terms of accuracy, the training accuracy increased quickly from below 50% and stabilized at approximately 95%, while the validation accuracy exceeded 90%, demonstrating the model’s overall ability to distinguish among different behavior categories.

(2) Precision and Recall

The model achieved high precision and recall on both the training and validation sets. On the training set, precision steadily increased from around 0.3 and eventually stabilized above 0.95. The precision on the validation set also remained above 0.88, indicating good robustness. Regarding recall, the training recall rose quickly and approached 0.96, while the validation recall remained stable at around 0.90, reflecting the model’s consistent ability to detect the majority of positive behavior instances.

(3) F1-Score Performance

**Figure 9:**
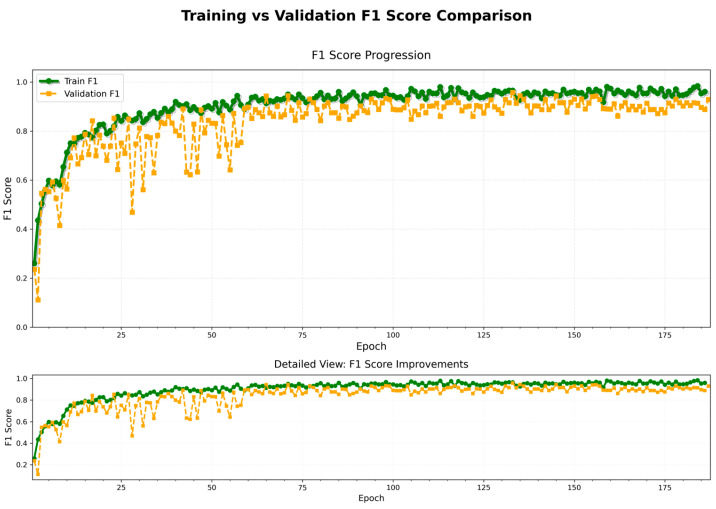
Trend of F1-score.

As illustrated in Figure 9, the F1-score progressively increased and converged during both the training and validation phases. The final F1-score on the training set stabilized at approximately 0.96, while the validation set maintained an F1-score above 0.91. This indicates that the model achieved a good balance between precision and recall, demonstrating stable and reliable recognition performance. A more detailed view further reveals that, with the increase in training epochs, the model exhibited a consistent improvement trend across all stages.

#### 3.2.2. Confusion Matrix Result

Normalized confusion matrix for the validation set.

**Figure 10:**
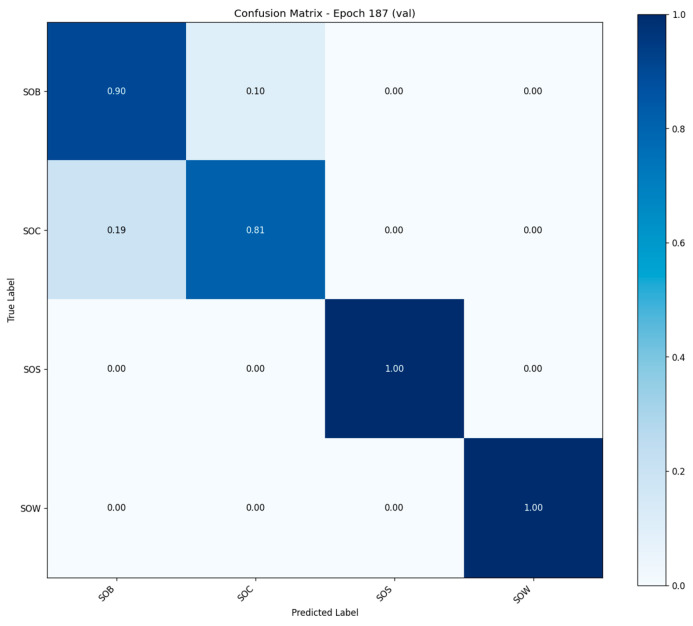
Confusion matrix of 3D-CNN Model.

The model achieved 100% classification accuracy on the SOW and SOS behavior categories, indicating a strong discriminative ability for these behaviors with clear temporal features. The accuracy for SOB behaviors reached 90%, while that for SOC was 81%, with some degree of mutual misclassification observed between the two. Overall, the diagonal elements dominate the matrix, suggesting that the model exhibits high prediction accuracy across all behavior classes and maintains well-defined classification boundaries (Figure 10).

#### 3.2.3. ROC Curves and AUC Values

The ROC curves of the model on both the training and validation sets exhibited smooth trajectories, consistently converging toward the top-left corner, indicating strong classification capability. On the training set, the AUC value rapidly increased from an initial 0.247 to a final value of 0.840. Similarly, the AUC on the validation set rose from 0.213 to 0.770, reflecting relatively large areas under the curve. These results further confirm the model’s discriminative ability in multi-class behavior recognition tasks.

### 3.3. CNN + TCN Model Performance

This section analyzes the performance of the behavior recognition model based on the fusion of CNN and TCN architectures in the task of sow behavior video classification.

The model’s classification accuracy, stability, and generalization ability are comprehensively evaluated through the analysis of performance metric trends during training and validation, confusion matrix, and ROC curves.

#### 3.3.1. Model Training Process 

**Figure 11:**
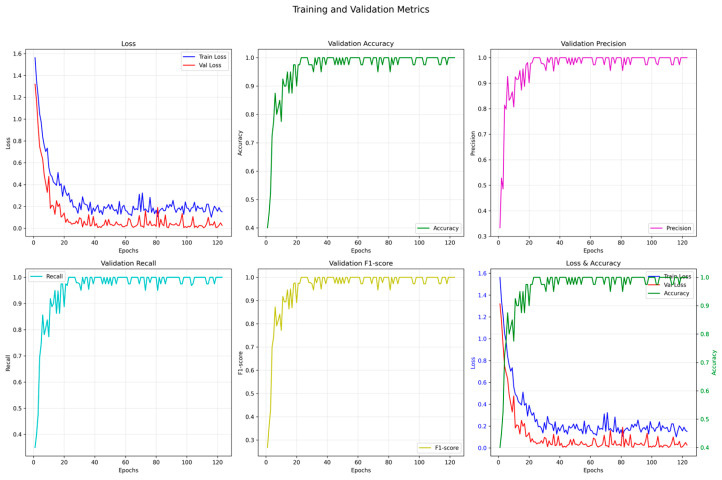
Model training process and performance metrics.

Figure 11 presents the key performance metrics of the CNN + TCN model over 120 training epochs, including training and validation loss, accuracy, precision, recall, and F1-score.

The training curves show a significant decline in loss, with the training loss rapidly decreasing from an initial value of 1.6 to below 0.2 within the first 20 epochs. The validation loss stabilizes after epoch 15, indicating fast convergence and a stable training process.

The classification performance is excellent: the validation accuracy remains consistently above 0.98 after epoch 30, while the recall and F1-score approach 1.0, suggesting balanced recognition capabilities across multiple behavior categories.

The precision, recall, and F1-score curves closely overlap, demonstrating that the model achieves strong overall classification performance when dealing with a dataset with balanced class distribution.

#### 3.3.2. Confusion Matrix Analysis

**Figure 12:**
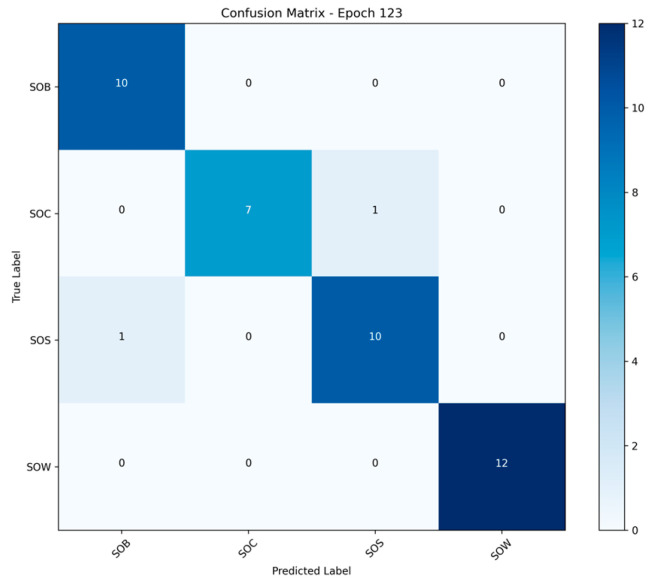
Confusion Matrix Analysis.

Figure 12 shows the confusion matrix of the CNN + TCN model on the test set after 123 training epochs. The classification results for each behavior category are as follows: all samples in the SOB (standing up) and SOW (standing during estrus ovulation) categories were correctly classified, achieving an accuracy of 100%, indicating the model’s extremely high discriminative power for these behaviors. In the SOC (mounting) category, one sample was misclassified as SOS (lying), and one SOS sample was misclassified as SOB. Overall, the model exhibits minor confusion among behaviors with relatively similar class boundaries. Most behavior samples are clearly concentrated along the main diagonal of the matrix, demonstrating strong overall classification capability and a low misclassification rate.

#### 3.3.3. ROC Curve and AUC Value Performance

**Figure 13:**
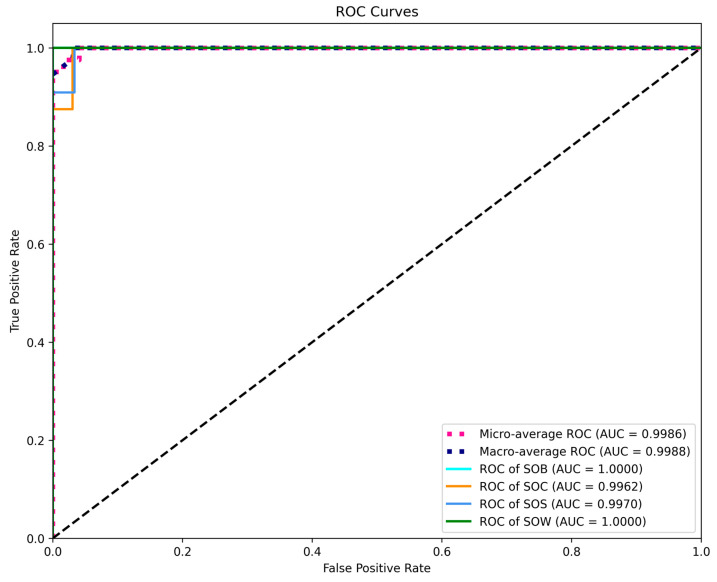
ROC Curve and AUC value performance.

Figure 13 presents the ROC curves of the CNN + TCN model for each behavior category on the test set. The results show that all categories achieved AUC values above 0.996, with SOB and SOW reaching 1.0000, and SOC and SOS achieving 0.9962 and 0.9970, respectively, indicating the model’s strong discriminative capability across different behavior types. The micro-average and macro-average AUC values were 0.9986 and 0.9988, respectively, reflecting the model’s superior overall performance and balanced recognition across categories, demonstrating excellent generalization ability.

In summary, the CNN + TCN model exhibited outstanding accuracy and stability in this study, with powerful capabilities in temporal behavior modeling, enabling the effective recognition of typical behaviors of sows under different physiological states.

## 4. Discussion

### 4.1. Overall Evaluation of CNN+LSTM Model

Based on the above experimental results, the CNN + LSTM model demonstrated excellent spatiotemporal modeling capabilities and overall classification performance in the sow behavior recognition tasks addressed in this study.

(1) Synergistic enhancement of spatial and temporal features

Compared with traditional 2D image modeling based solely on CNNs, the CNN + LSTM model effectively integrates inter-frame dynamic information through the introduction of a temporal sequence modeling module. This enables the model to extract not only spatial structural features but also capture the temporal patterns of behavior evolution. The results of the ROC curves (Figure 4) and confusion matrix (Figure 3) further validate the model’s stability and accuracy in action recognition tasks.

(2) Stable training and fast convergence

As shown in the training and validation loss and accuracy curves (Figure 2), the model rapidly improves key performance metrics within a relatively small number of epochs, with minimal oscillation throughout the training process. This suggests that the CNN component provides a solid foundation for spatial representation, while the LSTM effectively models temporal dependencies without introducing significant noise, indicating a well-structured architectural design.

(3) Robustness under small-sample conditions

The confusion matrix results indicate that the CNN + LSTM model maintains 100% classification accuracy even with limited sample sizes in certain classes (e.g., only 8 samples for the SOC category), highlighting its robustness and generalization capability. This suggests that the model is well-suited for real-world agricultural scenarios involving small-sample, unstructured data.

(4) Limitations and potential improvements

The model imposes a higher computational cost; compared to conventional CNNs, the LSTM introduces considerable time complexity, leading to prolonged training durations, especially in environments with limited GPU acceleration. Furthermore, the model’s responsiveness to short-duration, high-frequency behaviors is limited—LSTMs are better suited for modeling long temporal dependencies, and may underperform in capturing transient actions. Future improvements could consider integrating Temporal Convolutional Networks (TCN) or attention mechanisms to enhance the model’s sensitivity to instantaneous behaviors.

In addition, although the current dataset is relatively balanced, in larger-scale practical deployments, class imbalance may lead to insufficient learning for long-tailed categories. Addressing this issue may require incorporating advanced sampling strategies or class-weight adjustment mechanisms. In summary, the performance of the CNN + LSTM model in this study validates its effectiveness and feasibility as a baseline for temporal behavior recognition, laying a solid foundation for subsequent model optimization and deployment [27,28].

### 4.2. Comprehensive Assessment of the 3D-CNN Model

(1) Analysis of Model Convergence and Generalization Capability

As shown in the training and validation loss curves in Figure 5, the training loss decreases rapidly within the first 30 epochs and then gradually stabilizes. The validation loss follows a similar trend, ultimately converging to a relatively low level, indicating that the model exhibits good convergence behavior. Both training and validation accuracies exceed 90%, and no significant overfitting is observed on the validation set, reflecting the model’s strong generalization capability. Furthermore, the ROC curve in Figure 5 shows that the validation set achieves an AUC of 0.770, suggesting that the model has certain stability in distinguishing among behavior categories. However, a noticeable gap remains compared to the training set AUC of 0.840, indicating room for further improvement.

(2) Analysis of Precision–Recall Balance

Figure 5 illustrates the evolution of precision and recall during training and validation. Both metrics exhibit relatively synchronized growth throughout most epochs. Notably, during the first 50 epochs, both precision and recall increase rapidly and then converge gradually. Precision on the validation set stabilizes around 0.90, while recall is slightly lower but comparable. This suggests that the model does not suffer from an imbalanced tendency toward “high precision but low recall” or vice versa. The F1-score curve in Figure 6 further confirms this balance: the difference between training and validation F1-scores remains below 0.05 across all epochs, eventually stabilizing around 0.95 and 0.90, respectively. These results indicate that the model maintains consistent classification performance across categories and can effectively handle datasets with class imbalance.

(3) Analysis of Behavioral Recognition Difficulty and Confusion Phenomena

According to the confusion matrix in Figure 3, the SOS and SOW behavior categories are classified with 100% accuracy, indicating that the model achieves excellent discrimination for these stationary behavior patterns (e.g., lying down or standing still). In contrast, SOB (Standing on Boar) and SOC (Standing on Control) exhibit notable mutual misclassification, particularly with 19% of SOC samples being incorrectly predicted as SOB. This confusion may stem from two main causes: (1) the postural similarities and smooth transitions between these two behaviors, which result in overlapping visual features; and (2) the presence of short action clips that fail to capture the full temporal context, leading to inadequate feature representation. To address these issues, future work may consider incorporating attention mechanisms or using longer temporal window sequences to enhance the model’s ability to distinguish subtle behavioral differences.

(4) Multi-Metric Integration for Model Stability Evaluation

The ROC curve and AUC trends in Figure 5 provide a global perspective on model stability. The AUC value increases steadily from the early training epochs and begins to plateau after 50 epochs. Although the training set AUC reaches a peak of 0.84 and the validation set AUC is slightly lower at 0.77, both curves follow similar convergence trends, indicating no significant oscillations or performance degradation during training. When combined with the trends in precision, recall, and F1-score, it is evident that the model maintains a high degree of consistency across multiple evaluation metrics, demonstrating strong stability and robustness.

(5) Identified Limitations and Future Optimization Directions

Despite the model’s overall satisfactory performance, several limitations remain. First, the recognition accuracy for certain categories—such as SOC—is slightly lower, suggesting the need to enhance the temporal modeling capability for similar behaviors. Second, the validation AUC is consistently lower than that of the training set, indicating a mild tendency toward overfitting. Third, the recall increases relatively slowly during the early training stages, suggesting that model initialization or sample distribution may influence convergence efficiency. To address these issues, the following optimization strategies are proposed for future work: Incorporate more advanced temporal modeling architectures, such as Transformer or Bi-GRU, to improve contextual representation; Integrate visual attention mechanisms to enhance the representation of key behavior frames; Introduce class re-weighting schemes or focal loss to mitigate the impact of category imbalance.

### 4.3. Critical Evaluation of the CNN–TCN Model CNN+TCN Model

(1) Performance and Applicability Analysis of the CNN + TCN Model

This section provides an in-depth discussion on the training performance, classification capabilities, and applicability of the CNN + TCN model in the task of sow behavior recognition. Through an integrated analysis of training curves, confusion matrix, and ROC curves, the model’s advantages and limitations in extracting spatio-temporal coupled features are evaluated.

(2) Advantages in Model Performance

As illustrated by the trends in validation accuracy, precision, recall, and F1-score (Figure 1), the CNN + TCN model rapidly achieves a stable high-performance state and consistently maintains this level throughout the training process, indicating good convergence and generalization capability. Compared with traditional LSTM models, which suffer from low efficiency in long-sequence modeling, the convolutional structure and parallel computation design of TCN significantly improve both training efficiency and recognition accuracy.

The ROC analysis (Figure 10) further confirms the discriminative power of the model. The AUC values for all behavior classes are close to 1, suggesting that the model possesses excellent classification ability for distinct behavioral events (such as SOB and SOW, which exhibit clear action boundaries). In addition, both the micro-average and macro-average AUC scores are close to 1.0, demonstrating that the model handles sample imbalance effectively and avoids overfitting or neglecting specific classes. It can be seen that the convolutional structure efficiently captures both short-term and long-term dependencies, significantly improving recognition efficiency and accuracy. Based on validation performance metrics and ROC curve results, we further emphasize the model’s robustness and applicability in handling class imbalance and recognizing multi-stage behaviors.

(3) Classification Errors and Confusion Analysis

Despite the overall excellent classification performance, the confusion matrix (Figure 9) reveals minor misclassifications between the SOC and SOS categories. These behaviors may share visual similarity in certain postures or camera angles, especially under constrained viewpoints, short action durations, or occlusions, making localized confusion understandable. Nevertheless, the model achieves 100% classification accuracy for the SOW and SOB behaviors, indicating that the CNN + TCN structure is highly effective at capturing both gradually evolving patterns (e.g., SOW) and sudden motion changes (e.g., SOB), owing to its ability to model fine-grained local temporal features.

(4) Practicality and Deployment Potential

Based on the experimental results, the CNN + TCN architecture achieves high-precision recognition of key sow behaviors while maintaining relatively low model complexity, making it highly suitable for real-time applications and practical deployment. The model is well-adapted for behavior prediction tasks in continuous video streams, particularly in real-time monitoring systems within sow housing facilities. It can assist in reproductive management by improving the efficiency and accuracy of estrus detection. Considering the model’s training stability, ease of deployment, and sensitivity to sequential behavior patterns, CNN + TCN demonstrates a favorable balance of performance when compared to the other tested architectures (CNN + LSTM and 3D-CNN). Future work may consider incorporating attention mechanisms to enhance key-frame modeling strategies, thereby improving the model’s ability to filter out long segments of non-informative frames.

In summary, the three deep learning-based behavior recognition models and the integrated system platform proposed in this study provide an efficient and deployable solution for spatio-temporal modeling and intelligent recognition of sow estrus behaviors. In addition, many scholars have also carried out related studies in other livestock fields. For example, Hirata et al. conducted a cattle estrus detection study combining sensor information, and the results showed that this method has considerable advantages in estrus detection, indicating the potential of multi-sensor applications in pig and cattle production [29]. The research not only verifies the effectiveness of sequence modeling structures in the context of animal behavior perception but also lays a theoretical and practical foundation for the digitalization of reproductive behaviors and the intelligent management of pig farming. Future work will focus on enhancing the robustness of the models, improving the overall intelligence level of the system, and promoting its broader application in smart farming scenarios.

## 5. Conclusions

This study focuses on the automatic recognition of estrus behaviors in sows based on video sequence data, and systematically compares the performance and adaptability of three representative deep spatio-temporal modeling architectures: CNN + LSTM, 3D-CNN, and CNN + TCN. In addition, a real-time intelligent recognition system platform with a visual interface and closed-loop functionality—from behavior acquisition and feature modeling to result presentation—was developed and implemented.

Experimental results demonstrate that the CNN + LSTM model exhibits strong robustness in identifying behaviors with stable temporal characteristics (e.g., static standing, bar-biting), while the 3D-CNN model shows advantages in detecting short-term sudden behaviors (e.g., quick contact, shaking), though it presents certain limitations in long-sequence modeling. The CNN + TCN model achieves the best overall balance among classification accuracy, temporal modeling capability, and computational efficiency, delivering the highest comprehensive performance. All three models achieved high accuracy, recall, and F1-scores, confirming the feasibility and effectiveness of video-based deep learning methods for multi-class sow estrus behavior recognition. Furthermore, systematic analysis and visual interpretation of the classification results helped clarify the spatial and temporal characteristics distinguishing different behavior categories.

The proposed recognition system exhibits strong potential for real-world deployment. It supports non-contact, low-interference intelligent estrus detection in pig farming environments and is expected to significantly improve the efficiency and accuracy of estrus identification, thereby providing technical support for precision breeding management.

In summary, this study proposes a comprehensive deep recognition framework and evaluation system, offering a demonstrative pathway for the digital transformation of intelligent reproductive management in livestock farming. However, this study still has certain limitations. First, the experimental scenarios were limited, which may affect the model’s generalization ability in larger-scale and more complex environments. Second, the system relies heavily on computing resources and multi-source data collection equipment in practical deployment, which may restrict its large-scale application in ordinary farming settings in the short term. Future research will aim to introduce multimodal data fusion, lightweight deployment schemes, and integration mechanisms with reproductive management systems, to further enhance system intelligence and real-world applicability. Additional validation will focus on expanding the generalization capabilities of the proposed models to various farming scenarios, incorporating multisource sensing information (e.g., infrared, acoustic signals, physiological data) to enhance robustness and real-time performance. Ultimately, the goal is to integrate with production management systems to enable an intelligent closed-loop pipeline from estrus monitoring to breeding decision-making.

## Data Availability

The data presented in this study are available from the corresponding author on reasonable request.
